# Advances in Electrochemical Energy Devices Constructed with Tungsten Oxide-Based Nanomaterials

**DOI:** 10.3390/nano11030692

**Published:** 2021-03-10

**Authors:** Wenfang Han, Qian Shi, Renzong Hu

**Affiliations:** 1 Guangdong Provincial Key Laboratory of Advanced Energy Storage Materials, School of Materials Science and Engineering, South China University of Technology, Guangzhou 510640, China; wenfang.han@foxmail.com; 2 The Key Lab of Guangdong for Modern Surface Engineering Technology, National Engineering Laboratory for Modern Materials Surface Engineering Technology, Institute of New Materials, Guangdong Academy of Sciences, Guangzhou 510651, China

**Keywords:** tungsten oxides, energy storage devices, electrochromic devices, multifunctional devices

## Abstract

Tungsten oxide-based materials have drawn huge attention for their versatile uses to construct various energy storage devices. Particularly, their electrochromic devices and optically-changing devices are intensively studied in terms of energy-saving. Furthermore, based on close connections in the forms of device structure and working mechanisms between these two main applications, bifunctional devices of tungsten oxide-based materials with energy storage and optical change came into our view, and when solar cells are integrated, multifunctional devices are accessible. In this article, we have reviewed the latest developments of tungsten oxide-based nanostructured materials in various kinds of applications, and our focus falls on their energy-related uses, especially supercapacitors, lithium ion batteries, electrochromic devices, and their bifunctional and multifunctional devices. Additionally, other applications such as photochromic devices, sensors, and photocatalysts of tungsten oxide-based materials have also been mentioned. We hope this article can shed light on the related applications of tungsten oxide-based materials and inspire new possibilities for further uses.

## 1. Introduction

Energy exhaustion and environment deterioration has caused more and more scientific and public concern. To slow down the speed of resources running out and to ameliorate our living condition, turning to other inexhaustible energies including solar, wind, and tidal energy and employing high-efficiency devices to save energy have naturally become very important. Under the uncontrollable weather conditions, it is obviously challenging to get reliable and stable energy supply merely from inexhaustible energies. Therefore, those energy converting systems have to be used in conjunction with high-efficiency energy storage devices to store the converted energy [1,2]. As is known to us, supercapacitors [3,4] and lithium ion batteries [5] are two types of widely used efficient energy storage devices (ESDs). Moreover, electrochromic devices (ECDs) [6,7,8] are a well-known high-efficient application through controlling sunlight intensity and the amount of heat crossing it by changing transmittance.

Supercapacitors (SC) are one promising energy storage device for its unique advantages like high power density, ultra-long cycling life (over 10^5^ times), fast charging speed (within tens of seconds), and outstanding performance under low temperature [9]. There are two main types of SCs, electrical double-layer capacitors and pseudo-capacitors [3]. The former works basing on the centralization and decentralization of charge at the interface between electrode and electrolyte, while the latter operates mainly relying on Faradic reactions with their double layer capacitance making a relatively small contribution to total capacitance [10]. Typically, the capacitance of pseudo-capacitors is higher than that of electrical double-layer capacitors [11]. Lithium ion battery (LIB) is universally applied in portable electronic products and electric vehicles for their high energy density [12]. Now, the typical anode material in LIBs is graphite owing to its low cost, stable electrochemical properties, and good structural stability. Its theoretical specific capacity, 372 mA h g^−1^, however, is relatively low as energy consuming needs continually expand, thus limiting the further use of LIBs. Transitional oxide materials, such as tin oxides [13], cobalt oxides [14], and tungsten oxides [15] are considered potential alternatives to replace graphite owing to their higher specific capacity. 

Electrochromic (EC) materials can change their optical parameters including reflectance, refractive index, transmission, and emissivity when applied with a relatively low voltage (even smaller than 1 V) or an electric field [7], and this process is reversible when the polarity of the voltage or the electric field reverses. Because they possess this special property, ECDs are welcomed in smart windows, anti-dizziness rearview mirrors, display applications, and aerospace and military fields [16]. In particular, because energy consumption in buildings accounts for 40% of the global energy consumption [17], when they are used as smart windows, a large amount of energy can be saved due to their adjustable transmittance of sunlight. It has also been reported [18] that EC smart windows were superior to photovoltaic devices on energy savings. Moreover, in view of the fact that the color changing processes of ECDs also relate to ion insertion and extraction, an ECD can be considered as a transparent ESD.

The above three kinds of devices share similar device structure and operating principle. Furthermore, many transitional metal oxides, like MoO_3_ [19,20,21], MnO_2_ [22,23,24], and WO_3_ [15,25], can perform as the electrode material in these devices. Among them, tungsten oxides have large energy storage capacity that enable it to function as an electrode in ESDs, including SCs and LIBs, and it is also the most widely researched material in the EC field. When used as the electrode in SC, because the valence of W can be changed between +2 and +6, its theoretical specific capacity is 1112 F g^−1^ [26], much higher than the normally used double-layer capacitor’s carbon electrode material, and when as the anode in LIB, its theoretical specific capacity is 693 mA h g^−1^, nearly double that of graphite. In addition, they are also endowed with other advantages including high density, low cost, environmental friendliness, and nontoxicity. As in the EC field, the first EC phenomenon was found in tungsten oxide by Deb in the sixties [27]. Tungsten oxides are preferred for their short switching time, impressive color change, and electrochemical stability.

**Figure 1:**
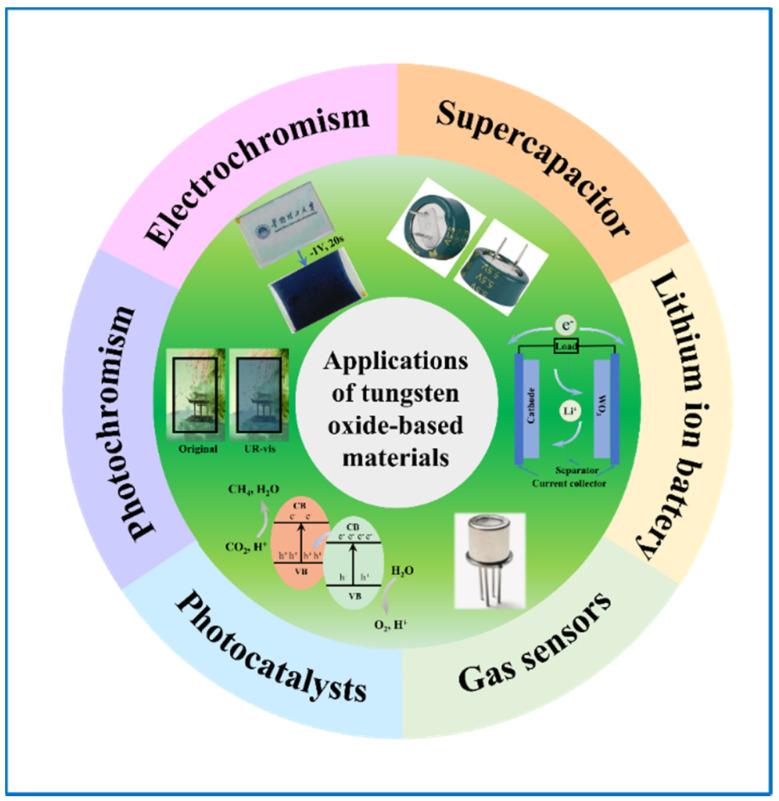
Applications of tungsten oxide-based materials for electronic devices.

Considering that ESDs and ECDs have several correlations, tungsten oxide electrochromic energy storage devices [28,29], whether it be electrochromic supercapacitors (ECSCs) or electrochromic batteries (ECBs), have also attracted much attention. We can get direct information about their working condition from color signals, bringing us great convenience and safety, or we can see it as a transparent battery and make good use of the energy stored in it, reducing electricity consumption. Moreover, these bifunctional devices can have more possibilities by integrating other parts, such as solar cells, so that self-powered systems are achieved [30,31]. Further, it can output power generated by the solar cells to effectively use the energy. In view of the versatile uses of tungsten oxide-based materials (Figure 1), there are many studies on them and some researchers have reviewed their developments in one or two specific fields, like R. Buch [32] in electrochromic, Dong [33] in photocatalysts, and V. Hariharan [34] in sensors. Yet, reviews focused on their comprehensive applications are still very rare. In this review, firstly (Section 2), we give a comprehensive introduction of the structures and uses of tungsten oxides, especially in energy storage devices, including tungsten oxide-based SCs, LIBs and ECs. Basic mechanisms and improving methods about tungsten oxides-based SCs and LIBs have been discussed (Section 3), following with the material and nanostructure design of tungsten oxides for EC applications (Section 4 and Section 5). Particularly, when used as an electrode in ECDs, their performances in near infrared (NIR) areas have been introduced. Furthermore, considering several connections like device structures, working principles, and materials involved, between ESDs and ECDs, tungsten oxide-based bifunctional devices are included in this part. Moreover, we mentioned the integration of solar cells in those bifunctional devices (Section 6). Finally, we provide a simple introduction to other applications including photochromism, photocatalyst, and gas sensors of tungsten oxide-based materials (Section 7), ending with a perspective on new functions and novel applications for smart, flexible, and especially self-powering tungsten oxide-based devices.

## 2. Energy Storage Mechanism of Tungsten Oxides

### 2.1. The Crystal Structure of Tungsten Oxides

**Figure 2:**
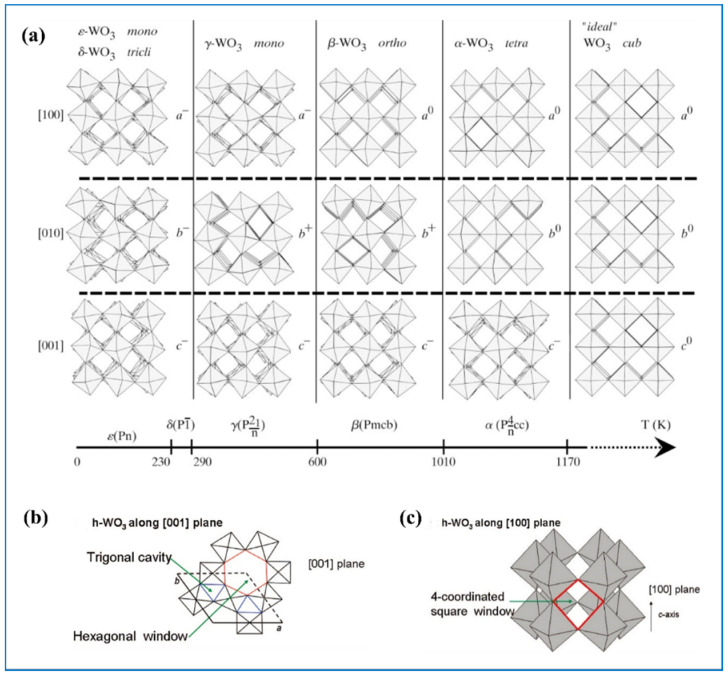
(**a**) Tilt patterns and stability temperature domains of the different polymorphs of WO_3_. Reproduced with permission from [41]. Copyright IUCr Journals, 2000. The structures of hexagon phase *h*-WO_3_ shown along (**b**) [001] plane and (**c**) [100] plane. Reproduced with permission from [40]. Copyright American Chemical Society, 2009.

In light of the fact that tungsten trioxide (WO_3_) is the most widely used of the tungsten oxides, we will mainly concentrate on WO_3_-based materials. The perfect WO_3_ is a kind of ReO_3_ type cubic structured material in which octahedral WO_6_ links each other by corner-sharing [25]. In a WO_6_ octahedron, the W atom lies at the center and the remaining six O atoms form the octahedral framework. As temperature and pressure change, the WO_6_ octahedron will tilt and rotate at certain angles, leading to the formation of several different phases: tetragonal phase, orthorhombic phase, monoclinic phase, triclinic phase, and cubic phase [35,36,37]. Figure 2a shows the phase transformation of WO_3_ as temperature changes. Within WO_3_, there are sites and tunnels between octahedrons so that atoms with small diameters such as H^+^, Li^+^, and K^+^ can transfer into WO_3_ and be stored. There is also another phase of WO_3_, hexagonal phase, that can be obtained from the hydrate-losing process of hydrated tungsten oxides [38]. As shown in Figure 2b,c, in the *a*-*b* plane of this phase, there are trigonal cavities and hexagonal windows. After stacking of the WO_6_ octahedron, trigonal and hexagonal tunnels along the *c* axel are formed [39,40]. These tunnels are conducive to the fast transfer of ions and electrons so the electrochemical activity of hexagonal tungsten oxides is better than the WO_3_ of other phases. The most common phases of tungsten trioxide are monoclinic phase (*m*-WO_3_), hexagon phase (*h*-WO_3_), and cubic phase (*c*-WO_3_).

Oxygen deficiencies are very common in the naturally existing WO_3_, causing the existence of substoichiometric tungsten oxides, WO_3−x_ (0 < x <1), in which the valence of W might be +3, +4, or +5 [42]. Among them, W_18_O_49_, W_20_O_58_, and W_24_O_68_ are the most common and the oxygen deficiencies within them can promote their conductivity. This characteristic renders WO_3_ an n-type semiconductor whose electric conductivity can be adjusted by controlling the amount of O vacancy in it [43,44,45].

Tungsten oxides made from liquid-related methods are often hydrated tungsten oxides before heat treatment, WO_3_·xH_2_O, mainly including WO_3_·0.33H_2_O, WO_3_·0.5H_2_O, WO_3_·H_2_O, and WO_3_·2H_2_O. The structure of WO_3_·xH_2_O is largely decided by the value of x. For example, the structure of WO_3_·H_2_O is that H_2_O lies in the gap between the layers of WO_6_ octahedrons [46,47], and the structure of WO_3_·2H_2_O is that in addition to the same kind of H_2_O molecule in WO_3_·H_2_O, another type of H_2_O molecule directly links to the tungsten atom at the bottom or top of the octahedron [48]. This structure is good for easy transport of ions and electrons, especially protons by means of the hydrogen bond network within them. Usually, hydrated tungsten oxides have much better conductivity than pure tungsten oxide, translating into enhanced electrochemical performance [47]. 

### 2.2. Phase Transformation in Tungsten Oxides toward Energy Storage

**Figure 3:**
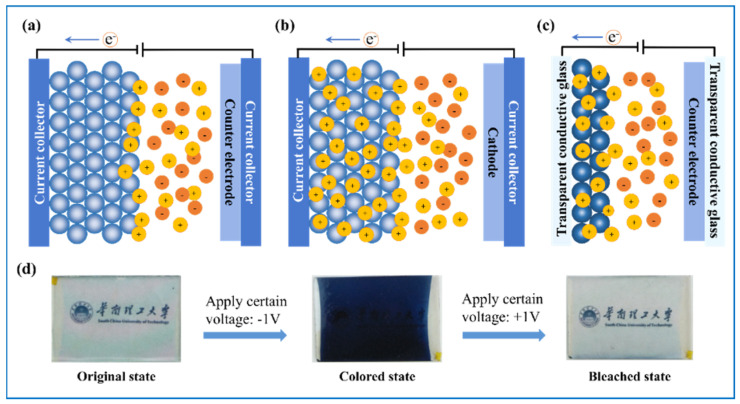
Structures and mechanisms of tungsten oxides working in (**a**) supercapacitor (SC), (**b**) lithium ion battery (LIB), and (**c**) electrochromic device (ECD). (**d**) Physical image of the color changing process of WO_3_.

As we mentioned above, SCs and LIBs are two typical ESDs, and ECDs can be seen as ESDs too. Here, we give a rough introduction and comparison between their working mechanisms and requirements of tungsten oxide electrodes. Figure 3 depicts their similarities and differences in terms of structures and mechanisms. SCs consist of five main parts, two electrodes, electrolyte, and current collectors at both electrodes (Figure 3a) [4]. As for pseudo-capacitors, they mainly rely on fast Faradic reactions on the interface between electrode and the electrolyte. Take WO_3_ as an example. When used as electrode material for pseudo-capacitors, WO_3_ works according to Equation (1) [49,50,51]:

WO_3_ + xM^+^ + xe^−^ = M_x_WO_3_ (1)

where M can be H, Li, Na, K, and other atoms or groups with small volumes. Different from tungsten oxides with other phases, *h*-WO_3_, except for this Faradic reaction, has another way of energy-storage by placing atoms in tunnels and cavities within its inner structure [52].

**Figure 4:**
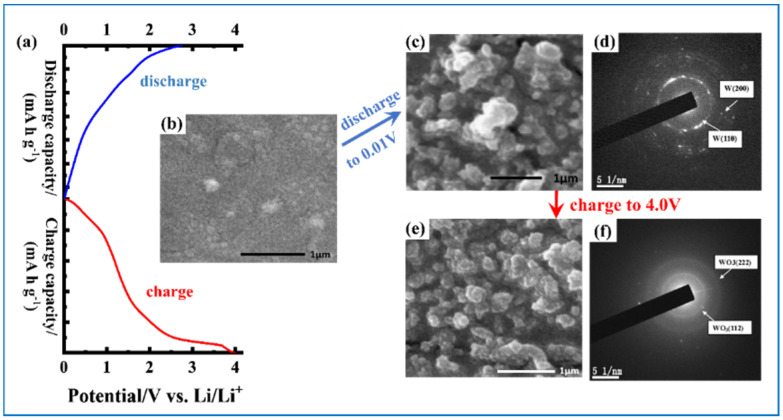
(**a**) Initial discharge and charge curves of WO_3_ thin film anode. (**b**) SEM image of as-deposited WO_3_ thin film; (**c**) SEM and (**d**) selected-area diffraction (SAED) images of WO_3_ thin film after initially discharged to 0.01 V; (**e**) SEM and (**f**) SAED images of WO_3_ thin film after first charged to 4.0 V. Adapted with permission from [15]. Copyright Elsevier, 2010.

LIB also shares the five-layer sandwich structure (Figure 3b). It works depending on Li^+^ ions’ movement between cathode and anode. Compared with that of pseudo-capacitors, the time needed for the charging and discharging process of LIBs is usually much longer because the redox reactions happen not only at the surface of the electrode but also in its deep bulk. Crystalline WO_3_ follows the conversion mechanism when as anode material in LIB, as presented in Equations (2) and (3) [15]. From the equations, we also get the theoretical capacity of WO_3_, 695 mA h g^−1^, when every W atom accommodates 6 Li^+^. Nevertheless, it is a double-edged sword because tungsten oxides suffer from large volume change at the same time, causing structural collapses and fast capacity decreases during cycling. Additionally, their low conductivity results in poor rate performance. As shown in Figure 4, the morphology and phase changes in the WO_3_ made by magnetron sputtering after initially fully discharged (at 0.01 V) and charged (at 4.0 V) have also been explored by scanning electron microscopy (SEM) and transition electron microscopy (TEM), revealing a large volume change of phase variation [15].

WO_3_ (crystalline) + 6Li^+^ + 6e^−^ ➝ W+3Li_2_O (initial discharging cycle) (2)

W + 3Li_2_O ⇆ WO_3_(nanocrystalline) + 6Li^+^ + 6e^−^ (3)

Usually, as shown in Figure 3c, to assemble a classic ECD, five components are needed, namely electrochromic layer, ion storage (IS) layer, counter electrode (CE) layer, and two transparent conducting (TC) layers. When the EC layer is WO_3_, the CE layer is usually anode EC materials such as nickel oxide [53,54], manganese dioxide [55], vanadium pentoxide [56], prussian blue [57,58], and some organic material like polyaniline (PANI) [59], etc., for they also present specific color change when the applied voltage changes. Their colors can have a synergy effect to strengthen the color of the ECD, or color superposition effect with the color of WO_3_ to enable the ECD to have multiple colors. For instance, when nickel oxide works as the CE layer, it turns brown when WO_3_ is colored, hence the color of ECD is deepened. When PANI performs the CE layer, the EC device can have four different colors (light green, green, light blue, and dark blue) as voltage regulates [59]. Sometimes, pure indium tin oxide (ITO) film [54,60] or fluorine doped tin oxide (FTO) film [61] can serve as CE layer as well, because they also have a large capacity of ions, leaving the ECD composed of four functional layers. However, it has been reported that an ECD with only single EC layer of tungsten oxide has relatively poorer EC performances than those of a complementary one [62,63].

WO_3_ is a cathodic EC material. Its color change from colorless to blue appears as a result of the insertion of ions and electrons when a negative voltage is applied. This process is reversible as the applied voltage turns positive. Thus, we can see ECDs as transparent ESDs. It has also been reported that these color changes happen owing to the change of band gap induced by the insertion and extraction of ions [64,65]. Figure 3d displays the optical image of the color changing process of WO_3_. This process can be implied as following [66]:

WO_3_ (bleached state) + xM^+^ + xe^−^ = M_x_WO_3_ (colored state) (4)

where M^+^ can be H^+^, Li^+^, K^+^, et al. 

As introduced above, it is found that the electrochemical reactions of WO_3_ in ECDs are similar to those in SCs and LIBs, which is very helpful for the development of integrated devices from WO_3_-based ESD and ECD. These three kinds of devices share the same sandwich structure. However, their different working mechanisms call for different requirements for tungsten oxide-based electrodes. Nevertheless, for all three kinds of devices, to get fast Faradic reactions, large specific surface area and good electrochemical conductivity of the tungsten oxides electrode are necessary.

## 3. Energy Storage Devices Based on Tungsten Oxides

### 3.1. WO_3_ Electrode Materials of Supercapacitors

WO_3_, with a theoretical capacitance of 1112 F g^−1^, is promising as an electrode material for pseudo-capacitors, but it also has drawbacks like unpleasant conductivity, poor rate performance, and less-satisfying cycling stability. The main improving methods can be divided into two parts, getting nanostructured single-phased tungsten oxide and getting multi-phased structures consisting of tungsten oxides with other materials such as carbon material, transitional oxides, and organic materials. Table 1 lists tungsten oxides as anode in SCs and their synthesizing methods and electrochemical performances, indicating that most of the WO_3_ nanostructures for the SCs were prepared by hydrothermal processes. 

#### 3.1.1. Single Phase WO_3_ Nanostructures

**Figure 5:**
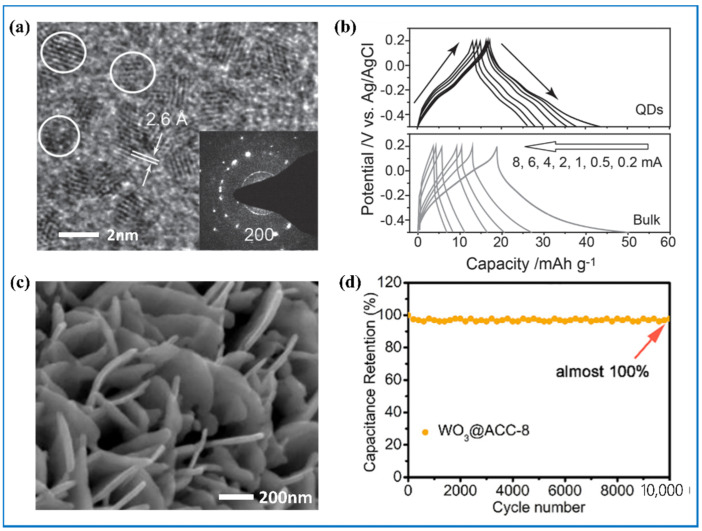
(**a**) High-resolution TEM image of as-prepared monodispersed tungsten oxide spherical quantum dots (QDs) with average sizes of 1.6 nm; (**b**) galvanostatic charge/discharge curves for QDs and bulk materials under currents of 0.2, 0.5, 1, 2, 4, 6, and 8 mA within potential from -0.5 to 0.2 V. Adapted with permission from [95]. Copyright John Wiley and Sons, 2014. High resolution SEM image (**c**) and cycling stability (**d**) of WO_3_ nanosheets. Adapted with permission from [69]. Copyright Elsevier, 2018.

It is well known that nanostructured materials have larger specific surface area by fining the size of materials, which makes them fully exposed to the electrolyte. The inner active materials can be very accessible to ions and electrons so that redox reactions can be accelerated. WO_3_-based nanostructures, including quantum dots [95] (Figure 5a) and nano particles, nanofibers [67], nanorods [68,72,73], nanotubes [70], nanochannels [81] and nanowires [74,75], nanoflakes [51], nanoplates and nanosheets [69] (Figure 5c), nanospheres [76], and nanoflowers have all been researched. Cong et al. [95] demonstrated that the WO_3_ quantum dots have better reversibility according to the more symmetric charge-discharge curve (Figure 5b) and more excellent rate performance. In particular, the WO_3_ nanosheets made by Yin et al. [69] can retain a capacity retention almost 100% after 10,000 cycles (Figure 5d). Huang et al. [96] got WO_3_ samples with different morphology by hydrothermal method: nanorods, nanoplates, and microspheres assembled of numerous nanorods. Among them, the ball cactus-like WO_3_ microspheres had larger specific surface area and more tunnels across these nanorods, translating into lower equivalent series resistance (R_s_) and excellent cycling stability, showing the best capacitive performance.

**Figure 6:**
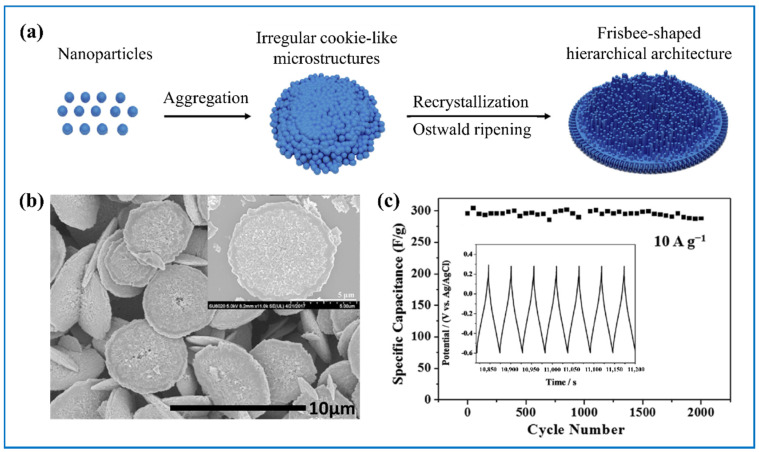
(**a**) Schematic illustration of the formation, (**b**) FE-SEM image, (**c**) charge-discharge curves at 0.5 A g^−1^, and (**c**) cycling test at 10 A g^−1^ of the frisbee-shaped crystalline h-WO_3_·0.28H_2_O. Adapted with permission from [77]. Copyright Elsevier, 2018.

Apart from the aforementioned nanostructures, there are also other more complex and interesting morphologies assembled by smaller nano-units. For example, Shao et al. [77] prepared frisbee-like WO_3_·nH_2_O microstructure assembled with numerous nanorods (Figure 6a,b). Thanks to this special micro/nano structure, it had high specific capacity of 391 F g^−1^ at 0.5 A g^−1^ and good rate capacity of 298 F g^−1^ under 10 A g^−1^. After 2000 charge-discharge cycles, its capacitance retention is around 100% (Figure 6c). By doping Pd, Gupta et al. [78] changed the morphology from nanosheets-assembled cabbage pure WO_3_ into nanobricks-assembled cauliflower Pd-doped WO_3_, achieving larger surface area. He et al. [50] fabricated a special furball-like microsphere, of which the core was assembled by a large number of nanorods and the shell was many other fluffy nanorods connecting each core, resulting in a porous 3D structure network. Notably, when used as the electrode in SC, even after 10,000 charge-discharge cycles, 93.4% of its initial capacitance was maintained. In addition, WO_3_ 3D nanorods array [71], cactus-like microspheres hierarchy 3D structure assembled by numerous nanorods [79,80], mesoporous pancake-like *h*-WO_3_ [52], and WO_3_·H_2_O flower-like hierarchical architecture composed of nanosheets [82] have also been reported, showing much enhanced performance in comparison to most WO_3_.

#### 3.1.2. Multi-Phased Tungsten Oxide Nanocomposites

Combining WO_3_ with other materials into composite is another direct way to achieve better performances in terms of good conductivity, high capacitance, and excellent stability. These composites obtained may possess the strengths of a single component and it is much easier to get a special structure that will further optimize its performances. 

Carbon materials have been frequently chosen for their attractive conductivity and low cost. Additionally, they are also used as an electrode in double-layer capacitors. The combination of double-layer capacitor material with pseudo-capacity material can have strengthened stability, capacitance, and rate performance. Di et al. [83] fabricated a feather duster-like carbon nanotube (CNT)@WO_3_ composite, in which WO_3_ nanosheet grows uniformly on the surface of CNT. After 8000 cycles of repeating cyclic voltammetry (CV) test at 100 mV s^−1^, this composite still retained 96.3% of its initial capacitance. Through a two-step hydrothermal method, Shinde et al. [49] made a composite in which multi-walled carbon nanotubes (MWCNTs) uniformly grew on the carbon cloth substrate and with WO_3_ nanorods growing on the MWCNTs. The prepared 3D structure had a large surface area and good structural stability. Chu et al. [84] synthesized WO_3_ nanoflower, well-coated with reduced graphene oxide (rGO) nanosheets. The capacity of WO_3_ and WO_3_-rGO composite were 127 F g^−1^ and 495 F g^−1^ respectively at current density of 1 A g^−1^, and when the current density was 5 A g^−1^, capacity of the composite was as high as 401 F g^−1^. These improvements were down to the shorter ion diffusion paths and 3D nanostructure of the composite. Liu et al. [85] embedded WO_3_ hybrid nanowires and nanoparticles in carbon aerogel and the electrode also showed a high capacity of 609 F g^−1^. Later, they used the same method dispersing size-selected WO_3_ nanoparticles in carbon aerogel and the results were also very pleasing [86]. 

Transition oxide materials such as V_2_O_5_, MnO_2_, CuO, and TiO_2_ are other typical electrode materials for pseudo-capacitors owing to their high capacitance and stability, and they are often used to form a composite with WO_3_. Shinde et al. [87] got a nanostructured composite of WO_3_ and MnO_2_, which had high capacity of 540 F g^−1^ at 2 mA cm^−2^ and good stability with 89% retention of initial capacitance after 2000 CV tests. Yuan’s group [88] also prepared nano-WO_3_*H_2_O/MnO_2_ composite with high capacity of 363 F g^−1^ at 0.5 A g^−1^. Periasamy et al. [89] reported a rod-shaped WO_3_-V_2_O_5_ composite prepared by microwave-assisted wet-chemical method. When in KOH electrolyte, its capacity was higher than pure WO_3_ by some distance. Moreover, it was noteworthy that after 5000 long cycles, the composite showed excellent capacity retention of 126% and had Coulombic efficiency of 100% up to 5000 cycles. In addition, WO_3_/TiO_2_ composites [90,91] and WO_3_@CuO composites [92] have been researched. 

As well as carbon materials and transition oxide materials, organic materials, especially conductive polymers, such as PANI, poly-3,4-ethylenedioxithiophene (PEDOT), and poly-pyrrole (PPy), are also preferred to combine with WO_3_ for their high conductivity, low cost, and easy fabrication. Zhuzhelskii’s group [93] dispersed WO_3_ in PEDOT. The porous PEDOT matrix ensured fast ion and electron transfer, thus promoting the electrochemical performance. Similarly, Das et al. [94] fabricated a WO_3_@PPy composite, in which WO_3_ nanostick is the core and PPy capsulated WO_3_. Owing to the high conductivity of PPy and the specific structure, shorter diffusion path length and greater stability were realized.

### 3.2. Tungsten Oxide-Based Materials as Anodes in Lithium Ion Battery

As mentioned before, when used as anode material in LIB, tungsten oxides suffer from structural collapses and fast capacity decreases during the charge-discharge cycling owing to the large volume change. Additionally, their low conductivity results in poor rate performance. So far, as listed in Table 2, some effective methods have been offered to improve the electrochemical performances of tungsten oxides. When O vacancies are introduced into tungsten oxides, its conductivity may be largely improved, and changing vacancy concentration to get increased conductivity has been tried. Nanostructures are not only adopted in SCs but also in LIBs. Moreover, adding carbon materials such as graphite, reduced graphite, and carbon nanotubes into tungsten oxide to get complex is also often adopted due to their high conductivity and structural stability.

#### 3.2.1. Non-Stoichiometric Tungsten Oxides

As introduced above, substoichiometric tungsten oxides are common in the natural world. O vacancies within them have a positive effect on the transport of electrons. In addition, WO_3_ is an n-type semiconductor, whose conductivity mainly depends on the concentration of free electrons in their conduction bands or, in other words, the concentration of donor within it [114]. By adjusting the ratio of W and O within tungsten oxides, the concentration of vacancies is changed accordingly, and its conductivity can drastically increase [115]. Thus, substoichiometric tungsten oxides such as W_18_O_49_ and W_20_O_58_ and tungsten oxides with naturally existing O vacancies are preferred. For example, Yoon et al. [97] prepared a mesoporous *m*-WO_3-x_ electrode. Though its initial Coulombic efficiency is only 53%, its reversible capacity reached 748 mA h g^−1^. Moreover, its electrical conductivity of 1.76 S cm^−1^ is also very competitive to mesoporous carbon materials (3.0 S cm^−1^). Li et al. [116] increased the density of O vacancy in tungsten oxide by annealing WO_3_ in N_2_ environment. The introduced O vacancy remarkably enhanced the conductivity of tungsten oxide, giving rise to excellent rate performance and reversibility of the electrode. 

**Figure 7:**
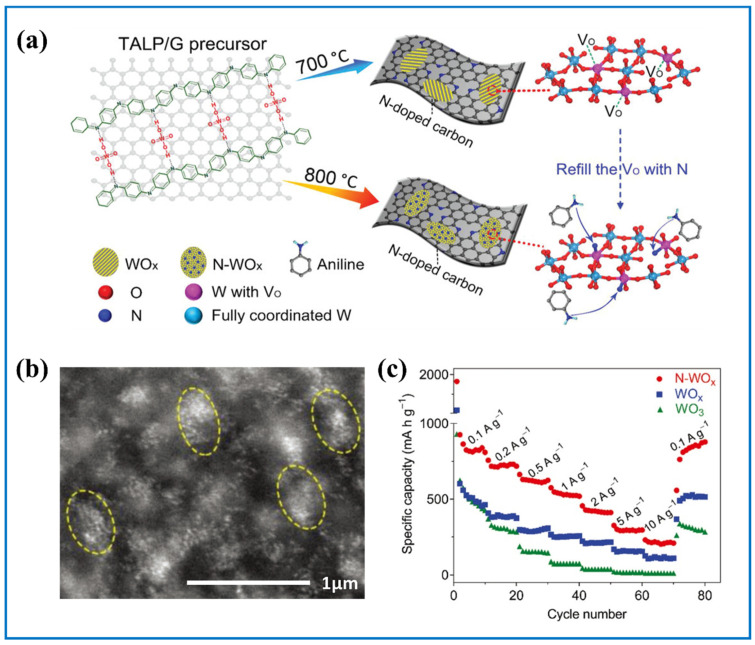
(**a**) Schematic illustration of the synthesis process for WO_x_ and N-WO_x_; (**b**) high-magnification high angle annular dark field scanning transmission electron microscopy (HAADF-STEM) image of N-WO_x_; (**c**) rate performance of WO_x_, N-WO_x_, and WO_3_ between 0.1 A g^−1^ to 10 A g^−1^. Adapted with permission from [98]. Copyright John Wiley and Sons, 2019.

Appropriate O vacancies concentration can translate into improved conductivity while excess O vacancies may be self-defeating. Sometimes we can also fill the O vacancy with other atoms that have similar diameter to O atom. Cui et al. [98] refilled O vacancies with N atom in WO_x_ (Figure 7a), transforming it into ultrafine disordered clusters (Figure 7b). The introduction of N offered many redox sites and facilitated the electrochemical kinetics, thus getting superior high-rate performance (Figure 7c). Aside from introducing O vacancies into tungsten oxides, excess O in tungsten oxides is also helpful because excess O can result in distortion of tunnels within tungsten oxides. Inamdar et al. [99] obtained tungsten oxide with excess O by adjusting the ratio of Ar to O_2_ in radiofrequency (RF) magnetron sputtering process. Results showed that the charge transfer resistance of WO_x_ under the gas ratio of 7:3 was tested to be 215 Ω, much lower compared with 370.8 Ω when the gas is pure Ar. They attributed this to the increased donor concentration induced by excess O in tungsten oxide. It is worth noting that the performance of tungsten oxide with excess O under high current density was also impressive.

#### 3.2.2. Nano-Structured Tungsten Oxides

**Figure 8:**
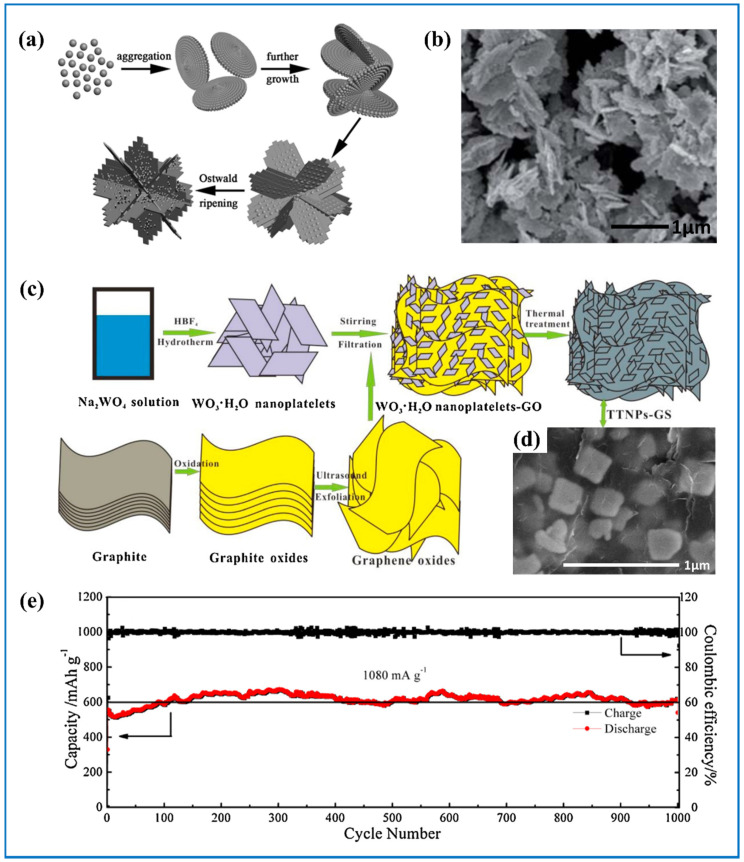
(**a**) Schematic illustration of the formation of hierarchical flower-like WO_3_·0.33H_2_O; (**b**) SEM image of WO_3_·0.33H_2_O. Adapted with permission from [101]. Copyright Society of Chemistry, 2011. (**c**) Schematic illustration of the formation of 3D hierarchical sandwich-type tungsten trioxide nanoplatelets and graphene (TTNPs-GS); (**d**) SEM overall appearance of single TTNPs-GS; (**e**) long cycling stability at 1080 mA g^−1^ for 1000 cycles of TTNPs-GS. Adapted with permission from [103]. Copyright Elsevier, 2016.

Through the sol-gel method, hydrothermal method, and template method, nano-structured tungsten oxides can be easily obtained. Wu et al. [70] made WO_3_ nanotube bundles by one-step hydrothermal and post-annealing process. Its initial specific discharge capacity and initial Coulombic efficiency were 871.9 mA h g^−1^ and 77.8%, respectively. Lim et al. [100] prepared WO_3_ nanocrystals and nanowires. Both samples showed high initial capacity of 867 and 954 mA h g^−1^ at 0.1C. For instance, after 100 cycles, the specific discharge capacity of WO_3_ nanowires retained 552 mA h g^−1^, and its average Coulombic efficiency was 97.2% during 2–100 cycles. Yang et al. [101] synthesized hierarchical flower-like WO_3_ using HCOOH as structure-directing agent in the hydrothermal method (Figure 8a). Every flower petal consisted of numerous nanorods (Figure 8b). At a current density of 100 mA g^−1^, its reversible capacity was 766 mA h g^−1^ after 50 cycles and still remained at 720 mA h g^−1^ even after 100 cycles. Additionally, under current density of 500 mA h g^−1^, its capacity was as high as 576.8 mA h g^−1^. All the results demonstrated good cycling and rate performance for the hierarchical flower-like WO_3_. Sasidharan et al. [102] used poly(styrene-b-[3-(methacryloylamino) propyl] trimethylammonium chloride-b-ethylene oxide) micelles (PS-PMAPTAC-PEO) as the template to produce WO_3_ hollow nanospheres. The whole triblock copolymer is composed of PS as its core, PMAPTAC as its shell and PEO as its corona. PMAPTAC can effectively bind with WO_4_^2+^ cations. After the following calcinations, the polymeric template is completely removed and the WO_3_ hollow nanosphere can be produced. 

#### 3.2.3. Tungsten Oxide-Carbon Composites

Introducing carbon materials into tungsten oxide to get composite can have several advantages. One is that the composite can integrate the advantages of both tungsten oxide and carbon material. The other is that it is more possible to form facile structures with high structure stability. Graphene is a flat monolayer based on single carbon atoms layer in a honeycomb lattice. This specific 2D structure gives it a super high theoretical specific surface area (2675 m^2^ g^−1^) [117] and offers high thermal and electronic conductivity. Zeng et al. [106] synthesized hierarchical sandwich composite consisting of WO_3_ nanoplatelets and graphene (Figure 8c). They added WO_3_*H_2_O nanoplatelets into the well-dispersed graphene oxide solution and then stirred the solution to form homogeneous suspension. Following that was the vacuum filtration process. After WO_3_*H_2_O nanoplatelets-GO was peeled from the membrane, it then underwent heat treatment and finally the WO_3_ nanoplatelets and graphene were obtained (Figure 8d). WO_3_ was embedded uniformly in the interlayer of graphene so that the electrode had stable cycling performance since the volume expansion of WO_3_ can be effectively relieved during cycling. At current density of 1080 mA g^−1^, its reversible capacity was kept at around 615 mA h g^−1^ after 1000 cycles (Figure 8e). Kim et al. [104] reported a 2D nanocomposite consisting of graphene nanosheets with WO_3_ nanoplates well scattered on it. After 50 cycles at 80 mA g^−1^, its capacity was 688.8 mA h g^−1^ compared with 555.2 mA h g^−1^ of pure WO_3_. Gu et al. [105] produced bamboo-like WO_3_ nanorods anchored on the N-doped 3D graphene frameworks. This composite can effectively bear the volume change and it provided higher conductivity for superior high-rate capability 

Reduced graphene oxide (rGO), usually obtained by reducing graphene oxide [118], is widely used to achieve better electrochemical performances of tungsten oxides. Dang et al. [106] successfully embedded WO_3_ nanoplates in a rGO matrix with a hydrothermal method followed by a heating treatment. Surprisingly, after 150 cycles under 100 mA g^−1^, its discharge capacity remained at 1005.7 mA h g^−1^, nearly twice that (565 mA h g^−1^) of pure WO_3_. The main reasons for this improvement can be ascribed to the fact that rGO can not only offer easier access for ions and electrons but also largely buffer the damage to its structure during cycling. Huang et al. [107] produced *h*-WO_3_ nanorods embedded in the rGO matrix doped with N and S. At a current density of 100 mA g^−1^, the composite possessed a specific discharge capacity of 1030.3 mA h g^−1^ at the first cycle and was down slightly to 816 mA h g^−1^ in the second cycle. Moreover, at a high current density of 1500 mA g^−1^, its specific discharge capacity was averaged at 196.1 mA h g^−1^ over 200 cycles. Park et al. [108] dispersed WO_3_ particles on 3D macroporous rGO frameworks. This special structure and rGO’s good conductivity jointly improved its rate capability and cycling stability.

Mesoporous carbon material is another kind of carbon material with high electrical and thermal conductivity, highly porous structure, and large specific surface area [119]. Wang et al. [109] dispersed ultrasmall WO_3_ nanocrystals into mesoporous carbon matrix. During the preparing process, the W species were limited by the carbon matrix, making the particle size of WO_3_ around 3 nm and high surface area of 157 m^2^ g^−1^ for the composite. After 100 cycles at current density of 100 mA g^−1^, its specific discharge capacity was 440 mA h g^−1^. Kim et al. [120] also achieved a nanocomposite in which WO_x_ nanoparticles were uniformly embedded in the mesoporous carbon matrix. Its main improvement was the lower polarization during the delithiation process owing to the high conductivity of mesoporous carbon matrix and shorter lithium on diffusion pathway.

**Figure 9:**
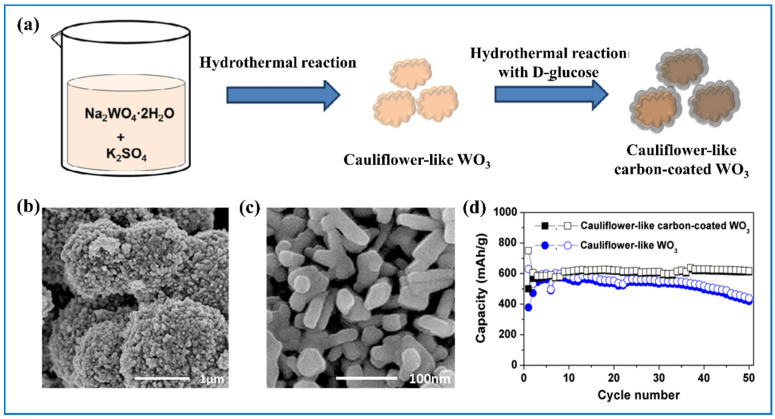
(**a**) Schematic illustration of the synthetic procedure, (**b**,**c**) SEM images for cauliflower-like carbon-coated WO_3_; (**d**) comparison of cycling performances of cauliflower-like WO_3_ and cauliflower-like carbon-coated WO_3_. Adapted with permission from [110]. Copyright Elsevier, 2014.

Aside from the above-mentioned carbon materials, amorphous carbon materials are also often used to achieve better electrochemical performance. For example, Yoon et al. [110] coated cauliflower-like WO_3_ with a thin layer of carbon (Figure 9a–c), which strengthened the electrochemical correlation between active WO_3_ and current collector and buffered the volume change as well. It showed much better cycling stability and rate performance than the pure WO_3_ (Figure 9d). Herdt et al. [111] made WO_3_ nanorod arrays encapsulated in a thin layer of carbon. After 200 cycles of charge-discharge at C/20, the vertical arrangement of nanorods were maintained, indicating the outstanding structural stability of this composite. In addition, Liu et al. [112] obtained a WO_3_*0.33H_2_O@C composite in which amorphous carbon was coated around WO_3_*0.33H_2_O. Interestingly, in his study, an appropriate amount of carbon coating can have positive effects while it can be self-defeating when the amount of carbon is in excess because it decreased the crystallinity of WO_3_·0.33H_2_O and sacrificed the capacity of the composite as well. Furthermore, Bao et al. [113] reported that the ultrathin WO_3-x_ nanoplate doped with carbon also showed excellent electrochemical performance. 

## 4. Electrochromic Applications

### 4.1. Tungsten Oxides as EC Electrode in Visible Light Area

Previous works about WO_3_ mostly concentrated on improving its EC performances in visible light spectrum area, with EC performances in the near infrared (NIR) and infrared (IR) spectrum area neglected. Their objects focused on getting WO_3_ EC films with wider optical modulation amplitudes, shorter response times, and higher coloration efficiency in the visible light area. To achieve these, efforts involving getting nanostructured tungsten oxides, porous structured tungsten oxides, and doped tungsten oxides were widely made. 

For example, tungsten oxide nanorods were produced by Khoo’s group [121] and its bleaching response time was significantly shortened to 4.5 s. Tungsten oxide nanobrick was synthesized by Kondalkar et al. [122], possessing fast switching response with coloration time and bleaching time of 6.9 and 9.7 s, respectively. Bhosale et al. [123] got WO_3_ nanoflowers film on the HCl-etched ITO substrate, its coloration efficiency and cycling stability had also been highly enhanced. Moreover, tungsten oxide quantum-dots, [124] nanowire arrays [125,126], nanobundles [127], nanosheets [128], nanoflakes [60], nanotrees [129,130], and nanoparticle-nanorod mixed structure [131] have been produced and tested to have enhanced EC performances.

**Figure 10:**
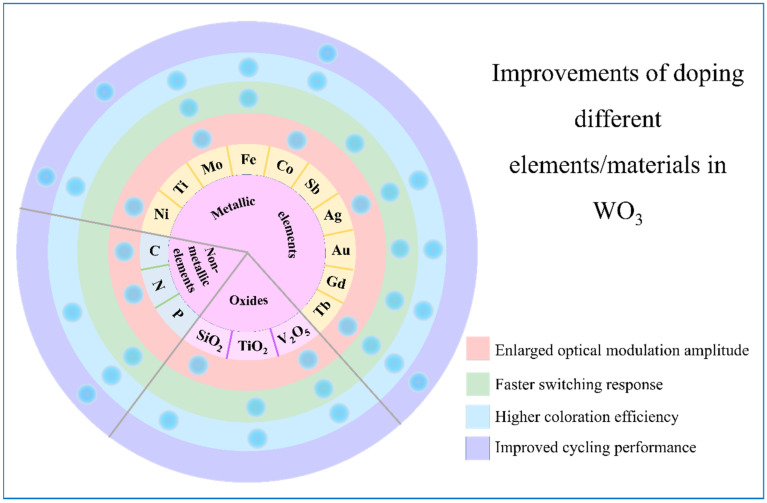
Improvements of WO_3_ film doped with different materials.

Doping tungsten oxides with an appropriate amount of other elements can have constructive effects on EC performances because the introduced deficiencies, morphology, and structure changes of the film adjust tungsten oxides’ crystallization and offer more ion storage sites. Peng et al. [132] got Ti-doped WO_3_ thin films that had less decay after 200 CV cycles than pure WO_3_. Koo et al. [133] added Fe into WO_3_ film. Compared with the switching time of 11.7 and 14.6 s for coloring and bleaching, respectively, of bare WO_3_, those were 7.2 and 2.2 s for 5% Fe-doped WO_3_. WO_3_ films doped with C [134], N [135], P [136], Ni [126,137], Mo [138,139], Co [140], Sb [141], Ag [142], Au [143], Gd [144], Tb [145], SiO_2_ [146], TiO_2_ [147], V_2_O_5_ [148], etc., have been reported before. Their positive effects on WO_3_ EC films are summarized in Figure 10.

### 4.2. Tungsten Oxides as EC Electrode in NIR Area

**Figure 11:**
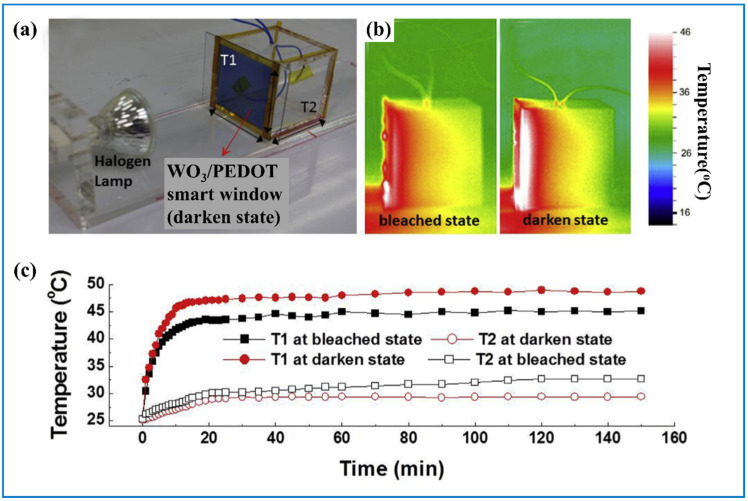
(**a**) Experiment setup for measurement of the capability of WO_3_/PH1000-based ECD on the modulation of solar heat; the thermal-imaging photography of the chamber under (**b**) bleached state and darkened state; (**c**) the temperature values of EC window (T1) and the back side of the chamber (T2) under bleached and darkened states. Adapted with permission from [150]. Copyright Elsevier, 2018.

Research shows that WO_3_ thin films have good control of the transmittance of not only visible light but also NIR and IR light so the temperature can be dynamically modulated, since IR light is the main resource of heat from the sun [36,48,62,149]. Jian et al. [150] reported that the WO_3_/PEDOT: PSS (poly (3,4-ethylenedioxythiophene):poly (styrene sulfonate) (PEDOT:PSS).) smart window can effectively reduce the heat across it. They detected the temperature of the back side of a small chamber that was assembled with an EC window as its front side (Figure 11a–c). When a halogen lamp worked as a radiation resource, it turned out that the temperature of the back side of the chamber was 3.3 °C lower as the EC window was darkened compared to the value as the EC window was bleached, demonstrating the EC film can effectively block heat (Figure 11d). Li et al. [151] made 1D W_18_O_49_ nanomaterials for NIR shielding. These films all had high transmittance in the visible light area. However, they did not explore the transmittance change during the color-changing process. Liu et al. [62] made a flexible ECD with transmission modulation of 63% between 760 and 1600 nm while they did not dig into the relationship between the transmittance of visible light and NIR light.

#### 4.2.1. Inverse Opal-Structured Tungsten Oxides

Inverse opal (IO) structure is a kind of 3D layered porous structure. It is favorable for its large specific surface area and artificially-ordered periodic layered configuration. This structure is often achieved by the template-assisted method, in which the template used is opal structure. After the material is deposited on the template, the template is removed, and the IO structure is obtained. Its large specific surface area was a result of the porosity obtained after the removal of template material so that electrolyte penetration can be bettered and the transmission of both electrons and ions are accelerated [146,147]. Owing to the periodicity and uniformity of the template, the final product also has a periodic and uniform structure, thus light reflection and refraction can be enhanced and it is beneficial for effective reduction of visible and NIR light transmittance [146,148,149].

**Figure 12:**
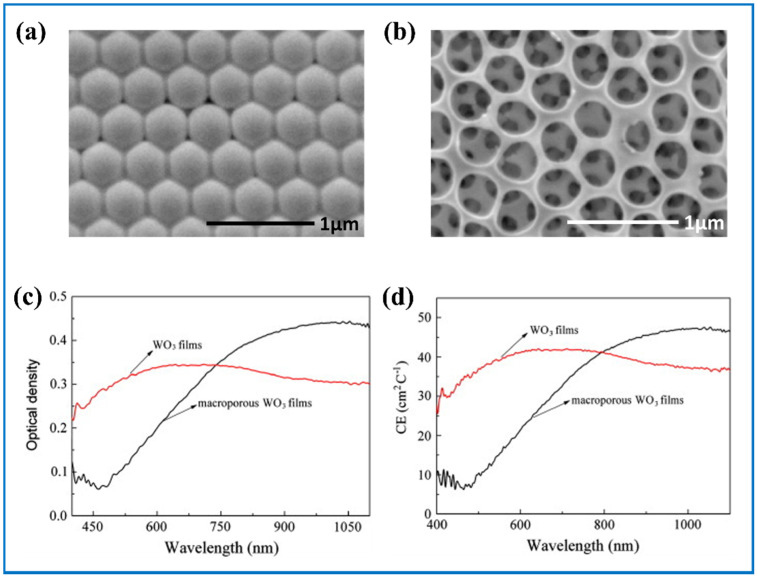
(**a**) SEM patterns of polystyrene (PS) template and (**b**) ordered macroporous WO_3_ films; (**c**) optical density and (**d**) coloration efficiency of WO_3_ films and ordered macroporous films. Adapted with permission from [152]. Copyright Elsevier, 2012.

Yang et al. [152] produced an ordered microporous tungsten oxide IO film using PS with different diameters as the template, followed by the process of removing PS through immersing the sample into tetrahydrofuran (THF), following which the porous tungsten oxide film was obtained (Figure 12a,b). Compared with the dense tungsten oxide film, this porous film showed high optical density and coloration efficiency in the NIR area (Figure 12c,d). They also found that smaller diameter of the porous and higher integration of ordered porous structure can translate into better EC performances. Later, a uniform SnO_2_-WO_3_ core-shell IO structure was reported by Nguyen et al. [153], aiming at good control of the transmission of NIR radiation without reduction in the transmission of visible light. They firstly got the SnO_2_ IO structure on the ITO coated base after removing PS. Following that was the electrodeposition of WO_3_. Finally, the specific core-shell SnO_2_-WO_3_ IO structure was successfully obtained. This EC film displayed high visible transparency of 70.3%, 67.1% at the wavelength of 400 nm at colored state, and blocked 62% of the NIR radiation at the same time. Later, adopting a similar method, Ling et al. [154] also made a TiO_2_–WO_3_ core–shell IO structure, which displayed well improved electrochromic performance in the NIR region as well.

#### 4.2.2. Dynamic Control of Visible and NIR Light of Tungsten Oxide ECDs

**Figure 13:**
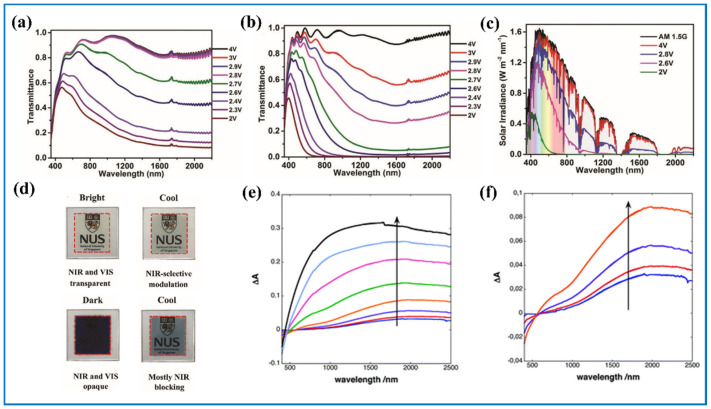
Optical transmittance spectra of the bulk *m*-WO_3_ film (**a**) and *m*-WO_3-x_ nanowires film (**b**); (**c**) solar irradiance spectra of *m*-WO_3-x_ nanowires films at 4, 2.8, 2.6 and 2 V; (**d**) physical photos of m-WO_3-x_ nanowires films on ITO glasses at 4 V, 2.8 V, 2.6 V, and 2 V (vs. Li^+^/Li). Adapted with permission from [155]. Copyright Royal Society of Chemistry, 2014. (**e**) Visible-NIR spectra showing the change in absorbance when a voltage is applied on the device, between the on (i.e., negative voltage, reduced tungsten oxide) and the off (i.e., positive voltage, oxidized tungsten oxide) states at 0.5, 0.7, 0.9, 1.1, 1.3, 1.5, 1.7, and 1.9 V; (**f**) zoom of the spectra obtained with lower voltages. Adapted with permission from [156]. Copyright American Chemical Society, 2012.

Different from the aforementioned studies that realized mere transmittance control of visible or NIR light, there were reports about dynamic transmittance control of both at the same time. In other words, when these materials are adopted in EC windows, three modes can be achieved, namely bright mode when both the transmittance of visible and NIR light are relatively high; cool mode when the transmittance of visible light is high while that of NIR light is relatively low; and dark mode when transmittance of both of them is low [155]. Zhang et al. [155] synthesized oxygen-deficient tungsten oxide nanowires that was able to control the transmittance of NIR and visible light independently. The film showed bright mode when the potential applied on active film was 4.0 V, cool mode when the potential was between 2.8 and 2.6 V, and dark mode when the potential was 2.0 V (Figure 13a–d). Reports have shown that amorphous and polycrystalline WO_3_ have different responses of light; that is, the light absorption peak of amorphous WO_3_ is more shifted into blue than that of crystalline WO_3_. Lia et al. [151] prepared WO_3_ films with hybrid phases to adjust the transmittance of visible and NIR light. It is reported that the WO_3_ flexible ECD they made had three different modes for the absorption of light in response to the applied voltage because an amorphous and a hexagonal phase of WO_3_ were both observed in the film. When the applied potential was lower than 1.1 V, the response of the NIR area was more active since the hexagonal portion of WO_3_ was at play, while under higher voltages, the response of visible area active for the amorphous portion was reduced (Figure 13e,f).

## 5. Electrochromic Energy Storage Devices (ECESDs)

As mentioned above, tungsten oxide is not only one of the candidates of electrode material in ESDs, including LIBs and SCs, but also an excellent material for ECDs. One device integrating these two functions has come into reality [157,158]. The idea of this integration rests chiefly on the following arguments. Firstly, ESDs share almost the same structure with ECDs, the sandwich structure [7,159]. Secondly, the working mechanisms of these two types of devices are also very similar. They both run relying on the redox reactions of ions in the electrolyte and active electrodes [160]. Thirdly, in the integrated device, tungsten oxide materials can be the electrode of the energy storage part and EC part at the same time [161,162]. Of course, aside from tungsten oxides, many other materials, especially some transitional metal oxides and conductive polymers, can be used as the active material in an integrated device as well. For example, bifunctional devices based on nickel oxide [163], vanadium pentoxide [164], and PANI [165,166] have all been reported before. These bifunctional devices can show us dynamic color signals, of which we can make good use to monitor how the device is running and to judge whether the device needs charging in case of energy cut-off. For another use, the energy stored in ECDs can be further used. Another relation is that the close charging and discharging time of SCs and switching time of EC devices also links them together. Consequently, integration of ECDs and SCs is more common compared with the integration of ECDs and batteries. However, in some situations where switching time does not matter that much, like smart windows and smart sunglasses, integrated ECBs can still have their place. Furthermore, as researchers are making dedicated efforts towards fast charging techniques of batteries, this gap is being filled in. In the following subsection, we will discuss tungsten oxides ECESDs from two main angles: research on single tungsten oxide electrode and exploration on complete ECSCs containing tungsten oxides as an electrode.

### 5.1. Tungsten Oxides Based ECESDs

**Figure 14:**
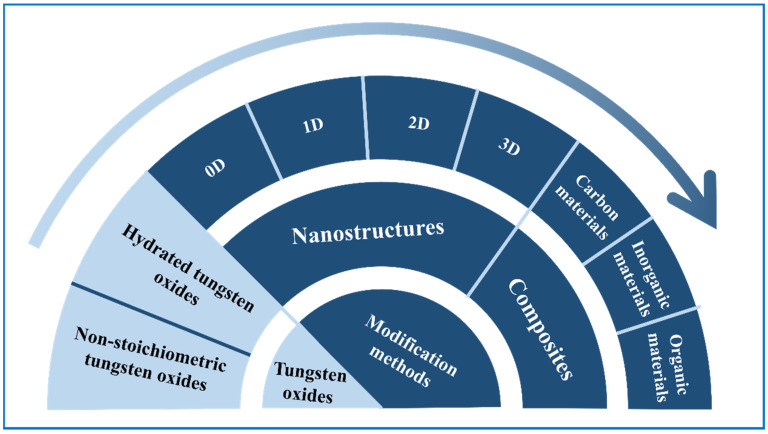
Main modification methods of tungsten oxide-based materials applied in electrochemical applications.

Approaches to enhance bifunctional performances of tungsten oxides electrode are very similar to those that improve electrochromic performance and energy storage performances. They are merely getting porous nanostructure, doping, and integrating tungsten oxide with other materials, especially organic materials (see Table 1, Table 2 and Table 3). Figure 14 sketches the main modification methods adopted by researchers. Most often, these methods are not adopted alone but two or three methods are adopted at the same time. Table 3 presents the electrochemical and EC performances of tungsten-based bifunctional electrodes.

Nanostructures can significantly facilitate the electrochemical activities of the electrode. For example, by hydrothermal method, He et al. [167] made different tungsten oxide nanostructures including nanospindles, nanopetals, nanosheets, and nanobricks. Among them, nanosheets had better EC performance of wider optical contrast, faster switching speed, higher coloration efficiency and capacitive performance of greater areal capacitance owing to its significantly increased active sites, and facilitated Li^+^ ions diffusion by the large surface area and porous structure. Soon after, their group [168] achieved WO_3_·H_2_O nanosheets by a one-step citric acid-assisted hydrothermal method with no need for a seed layer under a relatively low temperature of 90 °C. The film possessed large optical modulation of 79.0% at 633 nm and high areal capacitance of 43.30 mF cm^−2^. When the film was fully charged, its color turned blue. Wang et al. [161] fabricated a mesoporous WO_3_ film by a template assisted sol-gel method. It had faster switching time and higher storage capacity compared with microporous WO_3_ film owing to the reduced diffusion length and more exposed active sites in the mesoporous WO_3_ film. Later, their group [170] also made Nb-doped WO_3_ mesoporous cathode film by a sol-gel method. The results showed that slight doping of Nb enables the film to have wider optical modulation range, shorter switching speed, and higher capacitance because the introduction of the Nb element was accompanied by the introduction of O vacancy which increased the conductivity of the electrode. Other studies [162,171] reported that Mo-doped WO_3_ film had amorphous and porous structure, and was able to offer more channels for fast ion transfer and active sites for redox reaction.

**Figure 15:**
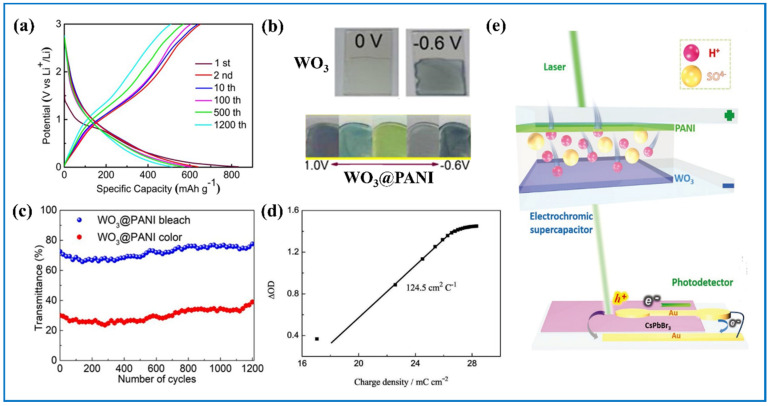
(**a**) The galvanostatic charge-discharge profiles of the urchin-like WO_3_@PANI electrode at current density of 0.2 A g^−1^; the photographs of (**b**) WO_3_ and WO_3_@PANI under different voltages; (**c**) the durability test of the urchin-like WO_3_@PANI composite film for 1200 cycles at a wavelength. Adapted with permission from [174]. Copyright Springer Nature, 2010. (**d**) Optical density variation with respect to the charge density at 633 nm. Adapted with permission from [177]. Copyright John Wiley and Sons, 2015. (**e**) Basic structure and mechanism of the in situ monitoring system composed of PANI//WO_3_ ECSCs and CsPbBr_3_ perovskite photodetector. Adapted with permission from [178]. Copyright John Wiley and Sons, 2019.

Many organic materials have large energy storage capacity and outstanding conductivity and are also good candidates for EC materials and have colorful color changes when voltage changes. When inorganic and organic materials are integrated together, some synergistic effects will occur. As an inorganic material, tungsten oxide has high electrochemical stability and long cycling life span but poor conductivity. When the former organic materials integrate with tungsten oxides, the final performance of the composite can be improved and the weaknesses of the single component can be attenuated. For example, the single-color display problem of tungsten oxide can be solved. One popular integrating idea is adding a material that can form donor-acceptor systems with tungsten oxide based on tungsten trioxide being an n-type semiconductor. It has been reported that this kind of donor-acceptor pair can have a strong combining force and it follows that the stability of the composite is highly strong so that the composite may have long life span [176]. On the other hand, the transport of ions and electrons will be facilitated. In addition, owing to the introduction of the organic material, the voltage window of the composite can also be widened. Many researchers have designed novel donor-acceptor pairs. Wei et al. [172] electropolymerized PANI onto the surface of tungsten oxide to form nanocomposite. They observed an obvious reduction of oxide peak currents in the CV curves of the nanocomposite compared to pure PANI owing to the donor-acceptor system. Results showed that not only did the nanocomposite have higher color efficiency (98.4 cm^2^ C^−1^) than pure tungsten oxide film (36.3 cm^2^ C^−1^) and pure PANI film (50.0 cm^2^ C^−1^), but also that its cycling performance was much more stable. Additionally, the potential window of the nanocomposite is wider than that of the pure PANI. Similarly, Nwanya et al. [173] also mentioned the slight reduction in CV curves of the WO_3_/PANI composite, which led to a more reversible redox reaction of the composite. Zhang et al. [174] used urchin-like WO_3_@PANI composite and showed that the composite electrode had better cycling stability than pure WO_3_ and maintained a reversible capacity of 516 mA h g^−1^ after 1200 charge-discharge cycles (Figure 15a,c). Furthermore, the colors of the composite were enriched, showing purple, green, yellow, gray, to blue as voltage changed (Figure 15b). Other organic materials have also been added to form this similar system. Guo’s group prepared the composite of WO_3_ and poly (indole-5-carboxylic acid) (P5ICA), where P5ICA is a p-doped material [158], and nanocomposites of WO_3_ and poly(5-formylindole) (P5FIn) where P5FIn acted as the p-doped semiconductor [175]. Both composite electrodes showed high capacitance and excellent cycling stability.

### 5.2. Quantitative Judgement of Energy Level Function of Tungsten Oxide Based ECESDs

Full tungsten oxide-based bifunctional devices provide a wider voltage window, possess larger capacity and have richer colors if an appropriate counter electrode is selected. Similar to tungsten oxide-based ECDs, the counter electrode of the device needs to be transparent. In order to get higher capacitance, a counter electrode with large capacitance is preferred. There have been reports [179,180] about the basic introduction of ECESDs, therefore, here, we just focus on an interesting characteristic of these devices.

There is a relation between energy storage level (ESL) of the tungsten oxide-based bifunctional device and its color, which is why we can get a direct qualitative message of the device’s state. Yet, in order to get precise judgement about the energy storage state, exploration of the accurate relationship between optical parameters and electrochemical parameters or getting help from other detectors is needed. We know that the more charges inserted into the EC film, the deeper the color displayed on the tungsten oxide-based bifunctional device and the higher ESL it has. It follows that we can get the qualitative monitoring by the relationship of transmittance and ESL. Coloration efficiency (CE) is the bridge between optical parameters and electrochemical parameters. Their relationship is expressed by the following equation [181,182]:

CE = (log (T_b_/T_c_))/Q (5)

where Q is the inserted charge density, T_b_ and T_c_ are transmittances of bleached state (fully discharged state) and colored state (fully charged state), respectively.

Because the CE of a device is almost fixed, we can get rough qualitative information of ESL through the relation of Q and log (T_b_/T_ESL_), as in the reports of Cai’s group [177] (Figure 15d), Bi’s group [183], and He’s group [167]. They painted the curve of optical density and charge density, though the curve is often used to calculate CE. At the same time, it reflected the rough correspondence between optical parameters and electrochemical parameters. Guo et al. [158] further changed the form of the formula into a more direct expression:

CE × Q_t_ × ESL = log (T_0_/T_ESL_) (6)

where Q_t_ is the total charge that can be stored in the device, T_0_ is the transmittance of the device fully discharged, and T_ESL_ is the real-time transmittance of the device. They achieved quantitative monitoring of device by getting the linear link of ESL and the optical density at the wavelength of 600 nm. Moreover, Sun et al. [178] successfully achieved the in-situ monitoring of the ESL of bifunctional device constantly and accurately with the help of an inorganic CsPbBr_3_ perovskite photodetector, based on the PANI//WO_3_ bifunctional ECESD they reported earlier (Figure 15e). This photodetector detects according to the change of response current to a green laser with the wavelength of 520 nm penetrated through the bifunctional device. It was so sensitive that even a voltage difference as small as 47.2 mV (charge variation of 0.33 mC) could be detected.

## 6. Solar Cell and Tungsten Oxide-Based EC Integrated Multifunctional Devices

The perfect use of tungsten oxide-based bifunctional ECESDs is the use of smart windows, as it can control the brightness and temperature in the room under a comfortable state by changing its transmittance of sunlight [184]. In addition, they can serve as a power to drive small electrical appliance like bulbs. Based on the position of this device, the introduction of a photovoltaic solar cell is natural and feasible. This idea is backed by the following reasons. Firstly, the open circuit voltage of the solar cells matches the relevantly low driving voltage of the tungsten oxide-based bifunctional device well [30,140]. Secondly, their positions in a building are not exclusive so that they can operate free of the influence of each other, thus achieving highly efficient energy use [185].

**Figure 16:**
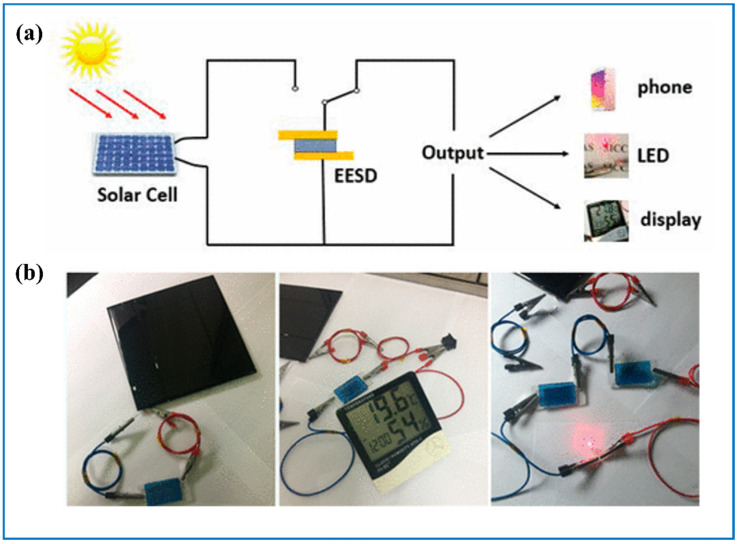
Integration of electrochromic energy storing device (ECESD) with silicon-based solar cells. (**a**) The circuit diagram of the smart operating system; (**b**) from left to right, ECESD is charging by solar cell, one ECESD can independently drive an LCD screen and two ECESDs in series can lighten a red LED. Adapted with permission from [31]. Copyright American Chemical Society, 2017.

**Figure 17:**
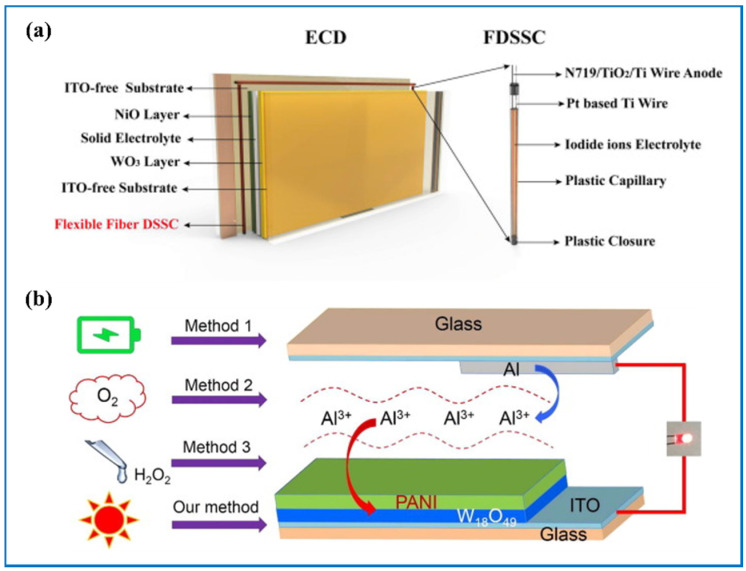
(**a**) Schematic diagram of the integrated system. Adapted with permission from [184]. Copyright Elsevier, 2019. (**b**) Schematic of charging routes for W_18_O_49_/PANI-EC battery. Adapted with permission from [187]. Copyright Elsevier, 2018.

Generally, the introduction of a solar self-powering part may have two main different forms. One is that the ECESD and the sunlight conversion device are separate and they are connected through wires outside the device. For example, Zhong’s group [59], Bi’s group [31] (Figure 16a,b), and Xie’s group [55] connected the tungsten oxide-based ECESD with silicon solar cell, Xia’s group [166] with perovskite solar cell, and Xie’s group [186] with dye-sensitized solar cells to achieve a self-powering system. Results showed that the bifunctional device can be fully charged without external power. Then, they can output energy in many forms including lighting up an LED bulb and the whole process can be continuously monitored by the color on the bifunctional device. Xu et al. [184] designed a new structure of the integrated system in which a tungsten oxide-based bifunctional device played as the glass and the fiber-shaped dye-sensitized solar cells served as the frame of the window so that space can be effectively used (Figure 17a). The shining point of this new system is that the numbers of the solar cells can be changed and when converting, they are free of the incidence angle since the frame was designed in a cylindrical configuration.

In addition to merely powering the bifunctional device, the whole self-powering smart window system can modulate its transmittance automatically in response to the changing weather condition. That is to say, when it is sunny, the solar cell can generate enough power to turn the window into dark state so that most of the sunlight is blocked to keep the room with a comfortable atmosphere and when it is cloudy, the generated power is not enough for the window to turn dark and light can partly penetrate through so that the room is still bright. This process was achieved in a report by Yun et al. [188] who took the solar cell as an automatic smart controller to modulate the transmittance of the tungsten-based bifunctional device according to the weather.

**Figure 18:**
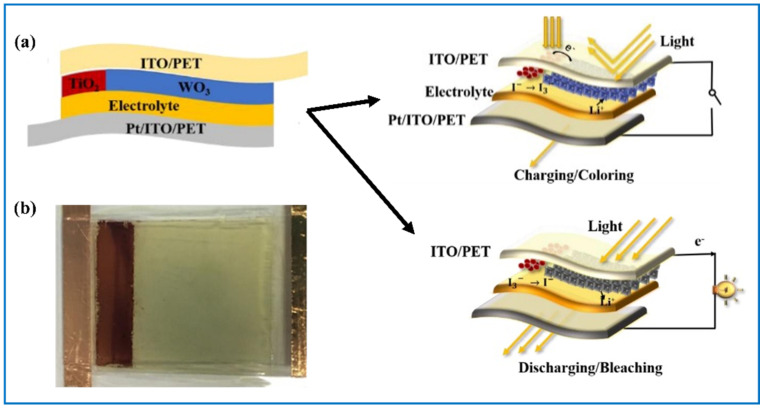
(**a**) Schematic diagrams of the trifunctional device; (**b**) photograph of the enlarged photoelectrochromic device (PECD) at the bleached state. Adapted with permission from [30].

The other type is that one device has three functions at the same time, namely solar-electricity conversion, EC and energy storage and powering, with no need for external wires. The EC part and PV part share the same electrolyte and typically, this kind of integrated method has to sacrifice a certain area of the window. For example, Zhang et al. [30] designed a trifunctional device, photoelectrochromic device (PECD), in which WO_3_ and TiO_2_ were both used as the anode and Pt was used as the counter electrode (Figure 18a,b). When the device is open circuit and exposed to sunlight, dye molecules in the electrolyte are excited and electron-hole pairs are generated. Then, the electrons are injected into the conduction band of TiO_2_ and later diffused to the ITO-PET substrate and then WO_3_, so WO_3_ film turns blue and the sunlight is blocked by the device as a result. The colored device can discharge when connected with electric applications. Based on tungsten oxide and PANI as the cathode and Al as the anode, Chang et al. [187] developed a multifunctional device that can be powered through two methods, by sunlight and by being exposed to the air (Figure 17b). Compared to the charging method without sunlight radiation, the time needed for the device to be fully charged is six times shorter.

## 7. Other Applications of Tungsten Oxides-Based Materials

Tungsten oxide is also an outstanding material in the photochromism field. When light is shed on tungsten oxide, electron-hole pairs are generated, leading to the change of optical absorption of them. What reflects directly to us is their color changes, and they can be applied as displays, smart windows, and optical signal processing. Wei et al. [189] indicated intrinsic WO_3_ with color change between yellow and blue when exposed to UV illumination. Zhang et al. [190] also synthesized tungsten oxide@poly(N-isopropyla- crylamide) composite spheres that had excellent response speed and impressed coloration efficiency. Finally, Wang et al. [191] have a comprehensive review about tungsten oxides-based photochromic materials.

The bandgap of WO_3_ in bulk is around 2.6 eV [192], so that it can absorb light with wavelengths up to 475 nm, indicating that it is a superior photocatalyst in the visible light area compared with TiO_2_ of which the band gap is 3.2 eV [33]. Currently, tungsten oxide-based materials as photocatalysts, are widely applied in CO_2_ photoreduction [193], air purification [194], and other fields. Specifically, in the photoelectrochemical cell (PEC) water-splitting field, tungsten oxides are also widely used. For example, J. Fu et al. [195] produced 2D/2D WO_3_/g-C_3_N_4_ and it showed good H_2_-production activity. Moreover, owing to its relative lower band gap, tungsten oxides are often applied to composite with TiO_2_ to expand the photoresponse of the composite into the visible light range [196,197]. For example, the composite of WO_3_/TiO_2_ made by Castro et al. [197] has a bandgap energy of 3.23 eV, which is between the values of pristine TiO_2_ and WO_3_, resulting in better light absorption at the visible range and higher efficiency of water splitting.

Another useful application of tungsten oxides is as gas sensors. As mentioned above, WO_3_ is a kind of n-type semiconductor of which the conductivity largely depends on the electrons within it. When it touches reductive gas, its conductivity will increase, while when it is exposed to oxidative gas, it decreases. By having the data of the conductivity changes, we can realize the detection of specific gases, like H_2_, H_2_S [198], CO, NH_3_ [199], NO_x_ [200,201], and some organic gases [202,203]. For instance, Liang et al. [203] produced ultra-thin WO_3_ nanosheets with dominant {002} crystal facets that have been proven to have improved xylene-sensing performance. Ji et al. [199] reported that tightly arranged nanosheet-assembled flower-like WO_3_ nanostructures showed better NH_3_-sensing performances compared with tightly arranged WO_3_ nanostructures.

## 8. Summary and Outlook

In summary, tungsten oxides are attractive for their numerous possibilities in various fields, particularly in energy storage like LIBs, SCs, and electrochromisms. Tungsten oxide-based multifunctional devices have also been widely explored based on the links between ESDs and ECDs. Furthermore, integration of solar converting system is a very effective way to realize a green application. Despite that the fact that much effort has been made in the research of tungsten oxides, there are many challenges to deal with. When tungsten oxides are used as the electrode in ESDs, low specific capacity, poor electric conductivity, and unsatisfied cycling stability still need to be improved. Furthermore, research about the tungsten oxide-based full ESDs are still rare. When they are applied in ECDs, their performance in the NIR area needs more attention. The bifunctional applications mentioned in this article also have weaknesses like single color, narrow voltage window, and low capacity, restricting their application to real uses. Apart from the aforementioned integration with solar cells, tungsten oxide-based devices call for new functions. For novel applications, such as flexible devices and self-powering methods by air or electrochemical reactions that have been reported before, improvements are still needed.

## Figures and Tables

**Table 1:** Electrochemical performances of different tungsten oxides-based electrodes in supercapacitors from literatures.

	Products and Structures	Synthesis Method	Electrochemical Performances		
			Potential Window, Reference Electrode, Electrolyte	Maximum Specific Capacity	Cycling Condition, Cycles, Capacity Retained
Single phase WO_3_ nanostructures	WO_3_ nanofibers [67]	Hydrothermal	−0.65–0 V vs. Ag/AgCl, H^+^	2 mA cm^−2^, 1.72 F cm^−2^	10 mV s^−1^, 6000 cycles, 79.1%
	WO_3-x_ nanorods [68]	Hydrothermal + annealing in hydrogen atmosphere	−10 V vs. SCE, 5 M LiCl	1 mA cm^−2^, 1.83 F cm^−2^	----, 10,000 cycles, 74.8%
	WO_3_ nanosheets [69]	Alcohol-thermal process	−1.0–0.5 V vs. Ag/AgCl, 0.5 M Na_2_SO_4_	5 mA cm^−2^, 0.659 F cm^−2^	---, 10,000 cycles, almost no decrease
	WO_3_ nanotubes [70]	Hydrothermal	−0.65–0.05 V vs. Ag/AgCl, 0.5 M H_2_SO_4_	3 mA cm^−2^, 2.58 F cm^−2^ 1 A g^−1^, 615.7 F g^−1^	2.5 A g^−1^, 6000 cycles, 85.11% (decreased from 496.4 to 422.5 F g^−1^)
	Furball-like WO_3_ microspheres [50]	Hydrothermal	−0.3–0.4 V vs. SCE, 2 M H_2_SO_4_	2 mA cm^−2^, 8.35 F cm^−2^ (=708.0 F g^−1^)	2 mA cm^−2^, 10,000 cycles, 93.4%
	WO_3_ nanorods array [71]	Hydrothermal	−0.6–0.3 V vs. Ag/AgCl, 2 M H_2_SO_4_	10 A cm^−2^, 5.21 F cm^−2^ 1 A g^−1^, 521 F g^−1^	3 A g^−1^, 2000 cycles, nearly 100%
	*h*-WO_3_ nanorods [72]	Hydrothermal	−0.7–0.2 V vs. SCE, 1 M H_2_SO_4_	5 mV s^−1^, 538 F g^−1^	100 mV s^−1^, 2000 CV cycles, 85%
	*h*-WO_3_ nanorods [73]	Hydrothermal	−0.5–0 V vs. SCE, 1 M H_2_SO_4_	0.35 A g^−1^, 694 F g^−1^; 0.93 A g^−1^, 484 F g^−1^	50 mV s^−1^, 2000 cycles, 87%
	WO_3_ Nanowires [74]	Solvothermal	−0.4–0.6 V vs. SCE, 0.1 M H_2_SO_4_	1 A g^−1^, 465 F g^−1^	----, 2000 cycles, 97.7%
	W_18_O_49_ Nanowires [75]	Solvothermal	−0.4–0.4 V vs. SCE, 1 M H_2_SO_4_	1 A g^−1^, 588.33 F g^−1^	1 A g^−1^, 5000 cycles, 88%
	*h*-WO_3_ nanoflake arrays [51]	Hydrothermal	1.0–1.8 V vs. Ag/AgCl, 1 M Na_2_SO_4_	0.5 A g^−1^, 538 F g^−1^	----, 5000 cycles, 95.5%
	WO_3_ nanospheres [76]	Hydrothermal	SCE, 2 M H_2_SO_4_	0.5 A g^−1^, 797.05 F g^−1^	5 A g^−1^, 2000 cycles, 100.47%
	Frisbee-like *h*-WO_3_*0.28H_2_O [77]	Hydrothermal	−0.6–0.3 V vs. Ag/AgCl, 1 M H_2_SO_4_	0.5 A g^−1^, 391 F g^−1^	10 A g^−1^, 2000 cycles, 100%
	3% Pd-doped WO_3_ nanobricks [78]	Hydrothermal	−0.7–0.1 V vs. Ag/AgCl, 1 M Na_2_SO_4_	0.5 A g^−1^, 33.34 F g^−1^	1 A g^−1^, 1100 cycles, 86.95%
	Cactus-like WO_3_ microspheres [79]	Hydrothermal	0.0–0.6 V vs. Ag/AgCl, 1 M Na_2_SO_4_	0.5 A g^−1^, 485 F g^−1^	1 A g^−1^, 5000 cycles, 93%
	Cactus-like WO_3_ microspheres [80]	Hydrothermal	−0.6–0.2 V vs. SCE, 2 M H_2_SO_4_	5 mV s^−1^, 970.26 F g^−1^	----, ----, ----
	Pancake-like *h*-WO_3_ [52]	Hydrothermal	−0.3–0.2 V vs. Ag/AgCl, 0.5 M H_2_SO_4_	0.37 A g^−1^, 605.5 F g^−1^; 7.5 A g^−1^, 276.0 F g^−1^	50 mV s^−1^, 4000 cycles, 110.2%
	WO_3_ nanochannels [81]	Electrochemical anodization	−0.8–0.5 V, 1 M Na_2_SO_4_	2 A cm^−3^, 397 F cm^−3^	10 A cm^−3^, 3500 cycles, 114%
WO_3_-carbon composites	Flower-like hierarchical WO_3_·H_2_O/reduced graphene oxide (rGO) [82]	Hydrothermal	−0.4–0.1 V vs. SCE, 1 M H_2_SO_4_	1 A g^−1^, 244 F g^−1^; 10 A g^−1^, 78 F g^−1^	4 A g^−1^, 900 cycles, 97%
	Feather duster-like carbon nanotube (CNT)@WO_3_ [83]	One-step solvothermal	−1–−0.3 V vs. Hg/HgSO_4_, 0.5 M H_2_SO_4_	0.5 A g^−1^ 496 F g^−1^; 10 A g^−1^, 407 F g^−1^	100 mV s^−1^, 8000 cycles, 196.3%
	Multi-walled carbon nanotubes-tungsten trioxide [49]	Hydrothermal	−0.6–0 V vs. SCE, 1 M LiClO_4_	2 mA cm^−2^, 429.6 F g^−1^ (1.55 F cm^−2^)	100 mV s^−1^, 5000 cycles, 94.3%
	WO_3_-rGO nanoflowers [84]	Hydrothermal	−0.4–0.3 V, 0.5 M H_2_SO_4_	1 A g^−1^, 495 F g^−1^	1 A g^−1^, 1000 cycles, 87.5%
	WO_3_ nanoparticles and nanowires in carbon aerogel [85]	----	−0.3–0.5 V vs. Ag/AgCl, 2 M H_2_SO_4_	5 mV s^−1^, 609 F g^−1^	50 mV s^−1^, 1000 cycles, 98%
	WO_3_ nanoparticles in carbon aerogel [86]	Solvent immersion + calcination	−0.3–0.5 V vs. Ag/AgCl, 2 M H_2_SO_4_	5 mV s^−1^, 1055 F g^−1^	500 mV s^−1^, 3000 cycles, 96% 50 mV s^−1^, 1000 cycling, 101%
WO_3_-transition oxide composites	Binder-free and additive-less WO_3_-MnO_2_ [87]	Hydrothermal	−0.6–0.6 V vs. SCE, 1 M Na_2_SO_4_	5 mV s^−1^, 609 F g^−1^ 2 mA cm^−2^, 540 F g^−1^	100 mV s^−1^, 2000 cycles, 89%
	WO_3_*H_2_O/MnO_2_ nanosheets [88]	Anodic deposition	−0.1–0.9 V vs. SCE, 0.5 M Na_2_SO_4_	0.5 A g^−1^, 363 F g^−1^	2 A g^−1^, 5000 cycles, 93.8%
	WO_3_–V_2_O_5_ nanocomposites [89]	Microwave assisted wet chemical route	KOH electrolyte	----, 173 F g^−1^	----, 5000 cycles, 126%
	2D WO_3_/TiO_2_ heterojunction [90]	Atomic layer deposition (ALD)	0.0–0.8 V vs. Ag/AgCl, 1 M H_2_SO_4_	1 A g^−1^, 625.53 F g^−1^	6 A g^−1^, 2000 cycles, 97.98%
	TiO_2_ nanoparticles-functionalized 2D WO_3_ film [91]	Two-step atomic layer deposition process + post-annealing	0.0–0.8 V vs. Ag/AgCl, 1 M H_2_SO_4_	1.5 A g^−1^, 342.5 F g^−1^ 30 A g^−1^, 285.3 F g^−1^	6 A g^−1^, 2000 cycles, 94.7%
	Porous WO_3_@CuO [92]	Template assisted method	0.0–0.5 V vs. SCE, 6 M KOH	1 A g^−1^, 284 F g^−1^	----, 1500 cycles, 85.2%
WO_3_-organic materials composites	PEDOT/WO_3_ [93]	Electrochemical deposition	−0.3–0.0 V vs. Ag/AgCl, (in 3 M NaCl), 0.5 M H_2_SO_4_	1.4 A g^−1^, 615 F g^−1^ 10 A g^−1^, 308 F g^−1^	----, ----,----
	WO_3_@PPy [94]	In situ oxidative polymerization process	−0.8–0.0 V vs. SCE, 2 M KOH	2 A g^−1^, 586 F g^−1^; 20 A g^−1^, 78% retained	5000 cycles, no significant changes in resistive property and morphology

**Table 2:** Electrochemical performances of different tungsten oxide-based electrodes in lithium battery from the literature.

	Products and Structures	Synthesis Method	Electrochemical Performances		
			Initial Efficiency	Voltage Window, Current Density, Capacity (Initial/Second)	Current Density/(mA/g), Cycles, Capacity Retained
Non-stochiometric tungsten oxides	m-WO_3-x_ [97]	Template method	53%	0–2.5 V, ---,748 mA h g^−1^ (1st)	---,---, ---
	N-WO_x_ [98]	Thermal annealing	52.2%	0–3.0 V, 100 mA g^−1^, 1760 mA h g^−1^ (1st); 817 mA h g^−1^ (2nd)	100 mA/g, 150 cycles, 954 mA h g^−1^ 10 A g^−1^, 4000 cycles, 228 mA h g^−1^
	Nanogranular WO_3_ with excess oxygen [99]	Magnetron sputtering	---	0–3.0 V, 100 mA g^−1^, 778.8 mA h g^−1^ (1st)	1 A g^−1^, 500 cycles, 217% retained
Nanostructured tungsten oxides	WO_3_ Nanotubes [70]	Hydrothermal	77.8%	0–3.0 V, 100 mA g^−1^, 1121.4 mA h g^−1^ (1st)	100 mA g^−1^, 200 cycles, 900 mA h g^−1^
	WO_3_ nanowires [100]	Hydrothermal	55.3%	0–3.0 V, 0.1 C, 954 mA h g^−1^ (1st)	0.1 C, 100 cycles, 552 mA h g^−1^
	Flower-like *h*-WO_3_ [101]	Hydrothermal + calcination	---	0–3.0 V, 100 mA g^−1^, 2086.4 mA h g^−1^ (1st)	100 mA g^−1^, 100 cycles, 720.5 mA h g^−1^
	WO_3_ hollow nanospheres [102]	Soft template assisted method	74.0%	0–3.0 V, 0.2 C, 1054 mA h g^−1^ (1st)	0.2 C, 100 cycles, 294 mA h g^−1^
Carbon-tungsten oxides composites	3D sandwich-type architecture with 2D WO_3_ nanoplatelets and 2D GS [103]	Hydrothermal + ultrasonic stirring + thermal treatment	71.8%	0–3.0 V, 72 mA g^−1^, 1262 mA h g^−1^ (1st)	1800 mA g^−1^, 500 cycles, 397 mA h g^−1^
	WO_3_ nanoplates and graphene nanosheets 2D nanocomposites [104]	Hydrothermal + heating process	---	---, ---, ---	400 mA g^−1^, 50 cycles, 455 mA h g^−1^ (64.3% retained)
	Bamboo-like WO_3_ nanorods anchored on 3D nitrogen-doped graphene frameworks [105]	Hydrothermal + heating process	64.5%	0–3.0 V, 1280 mA h g^−1^ (1st)	80 mA/g, 100 cycles, 828 mA h g^−1^ (73.8% retained)
	WO_3_ nanosheet@rGO square particles [106]	Hydrothermal	87.9%	0–3.0 V, 100 mA g^−1^, 1143 mA h g^−1^ (1st)	100 mA g^−1^, 150 cycles, 1005.7 mA h/g
	*h*-WO_3_ nanorods embedded into nitrogen, sulfur co-doped rGO nanosheets (54 wt %) [107]	Ultrasonic processing + hydrothermal	---	0–3.0 V, 100 mA g^−1^, 1030 mA h g^−1^ (1st), 816.3 mA h g^−1^ (2nd)	1500 mA g^−1^, 200 cycles, 196 mA h g^−1^
	WO_3_ particles deposited on 3D macroporous rGO frameworks [108]	Hydrothermal + freeze-drying	57.23%	0–3.0 V, 50 mA g^−1^, 1120 mA h g^−1^ (1st), 719 mA h g^−1^ (2nd)	150 mA g^−1^, 100 cycles, 487 mA h g^−1^ (~99% retained)
	Ordered mesoporous carbon/WO_3_ [109]	Evaporation induced self-assembly	56.2%	0–3.0 V, 100 A g^−1^, 1275 mA h g^−1^ (1st), 712 mA h g^−1^ (2nd)	100 mA/g, 100 cycles, 440 mA h g^−1^
	Cauliflower-like WO_3_ decorated with carbon [110]	Hydrothermal + firing	67%	0–3.0 V, 50 mA g^−1^, 750 mA h g^−1^ (1st) and 500 mA h g^−1^ (2nd)	50 mA/g, 50 cycles, 650 mA h g^−1^ (~Li_5.5_WO_3_)
	Carbon-coated 3D WO_3_ [111]	Template assisted process	60.1%	0–3.0 V, C/20, 10,791 mA h g^−1^ (1st), 649 mA h g^−1^ (2nd)	---, 500 cycles, 253 mA h g^−1^
	WO_3_*0.33H_2_O@C nanoparticles [112]	Low temperature combustion	46.1%	0–3.0 V, 100 mA g^−1^, 1543 mA h g^−1^ (1st)	100 mA g^−1^, 200 cycles, 816 mA h g^−1^
	Ultrathin WO_3−x_/C nanosheets [113]	Acid-assisted one-pot process	39.4%	0–3.0 V, 200 mA g^−1^, 1866 mA h g^−1^ (1st), 893 mA h g^−1^ (2nd)	200 mA g^−1^, 100 cycles, 662 mA h g^−1^

**Table 3:** Performance of tungsten oxide-based electrochromic energy storage electrodes from literatures.

Products and Structures	Method	Electro-Chromic Energy Storage Type	Electrochromic Performances			Energy Storage Capacity (C)	Cycling Performances
			Optical Transmittance Modulation (▲T)	Switching Time (t_c_, t_b_)/s	Color Efficiency/(cm^2^/C)		
WO_3_ nanosheets [167]	Hydrothermal	ECSC	64.5% (633 nm)	6.6, 3.8	48.9	14.9 mF/cm^2^	1000 cycles, ▲T 83.7% retained C 84.5% retained
WO_3_·H_2_O nanosheet [168]	Hydrothermal	ECSC	79.0% (633 nm)	10.1, 6.1	42.6	43.30 mF/cm^2^	2000 cycles, ▲T 87.8% retained
Oxygen-rich nanograin WO_3_ [169]	Oblique-angle sputtering	ECSC	82% (630 nm)	---, ---	~170	0.25 A g^−1^, 228 F g^−1^	2000 cycles, C 75% retained
Mesoporous WO_3_ film [161]	Sol-gel	ECB	75.6% (633 nm)	2.4, 1.2	79.7	75.3 m A h g^−1^	------
Nb-doped WO_3_ film [170]	Sol-gel	ECB	61.7% (633 nm)	3.6, 2.1	49.7	74.4 m A h g^−1^	1000 cycles, ▲T 76.2% retained, C 75.8% retained
Mo-doped WO_3_ nanowire arrays [171]	Hydrothermal	ECB	56.7% (750 nm), 83.0% (1600 nm)	3.2, 2.6 (750 nm)	123.5 (750 nm)	55.89 m A h g^−1^	3500 cycles, ▲T 57.3% retained; 4 A/g, 5500 cycles, C 38.2% retained
Amorphous Mo-doped WO_3_ films [162]	Electrodeposition	ECSC	83.3% (633 nm)	2.1, 2.0	86.1	0.25 mA/cm^2^, 117.1 mF/cm^2^ (334.6 m F g^−1^)	4000 s, ▲T no obvious change 1500 cycles, C 83% retained
PANI/WO_3_ nanocomposite [172]	Electropolymerization + annealing	ECSC	35.3% (633 nm)	13.6, 9.9	98.4	5 mV/s, 0.025 F/cm^2^	1000 cycles, charge density did not change too much
WO_3_/PANI nanocomposite [173]	Chemical bath	ECSC	Color changes: brownish green-transparent-light green-brownish green	---, ---	---	0.02 mA/cm^2^, 4.1 mF/cm^2^	800 cycles, C 38% retained
Urchin-like WO3@PANI [174]	Solvothermal + electropolymerization	ECB	45% (700 nm)	1.9, 1.5	---	---, 831 mA h g^−1^	1200 cycles, 516 mA h/g
Honeycombed porous poly(5-formylindole)/WO_3_ nanocomposites [175]	Hydrothermal + electrochemical polymerization	ECSC	26% (505 nm); 46% (745 nm)	---, ---	137	---, 34.1 mF/cm^2^	5000 cycles, C 93% retained

## Data Availability

All authors ensure that data shared are in accordance with consent provided by participants on the use of confidential data.
